# M2 macrophage infiltration associated with *CXCL8* predicts grade 4 prognosis and differentiates glioma grades

**DOI:** 10.1007/s12672-025-03982-2

**Published:** 2025-12-23

**Authors:** Wei-Hsiu Liu, Cheng-Chin Lee, Yu-Jia Chang, Ai-Wei Lee, Chien-Yu Huang, Tuan Ngoc Anh Vo, Yung-Fu Wu, Jang-Chun Lin

**Affiliations:** 1 Department of Neurological Surgery, Tri-Service General Hospital, National Defense Medical University, Taipei, 11490 Taiwan, ROC; 2 Department of Surgery, School of Medicine, National Defense Medical University, Taipei, Taiwan, ROC; 3 Graduate Institute of Medical Sciences, College of Medicine, Taipei Medical University, Taipei, Taiwan, ROC; 4 Graduate Institute of Clinical Medicine, College of Medicine, Taipei Medical University, Taipei, Taiwan, ROC; 5 Cell Physiology and Molecular Image Research Center, Wan Fang Hospital, Taipei Medical University, Taipei, Taiwan, ROC; 6 Department of Pathology, Wan Fang Hospital, Taipei Medical University, Taipei, Taiwan, ROC; 7 Department of Anatomy and Cell Biology, School of Medicine, College of Medicine, Taipei Medical University, Taipei, Taiwan, ROC; 8 School of Medicine, National Tsing Hua University, Hsinchu, 300044 Taiwan, ROC; 9 Department of Manufacturing, Faculty of Mechanical Engineering, Ho Chi Minh City University of Technology (HCMUT), VNU-HCM, Ho Chi Minh City, Vietnam; 10 Department of Medical Research, Tri-Service General Hospital, National Defense Medical University, Taipei, Taiwan, ROC; 11 Department of Radiation Oncology, Shuang Ho Hospital, Taipei Medical University, New Taipei City, 23561 Taiwan, ROC; 12 Department of Radiology, School of Medicine, College of Medicine, Taipei Medical University, No. 250 Wu-Hsing Street, Taipei, 110 Taiwan, ROC

**Keywords:** Glioma, CXCL8/IL8, M2 macrophage, Prognostic biomarker

## Abstract

**Background:**

This study aims to improve glioma-WHO grading by identifying biomarkers distinguishing Gr. 4 from Gr. 3 gliomas and exploring their immune-related prognostic significance.

**Methods:**

We analyzed differentially expressed genes (DEGs) between Gr. 3 and Gr. 4 gliomas using the GSE4290 (31 Gr. 3, 77 Gr. 4) and GSE109857 (34 Gr. 3, 89 Gr. 4) datasets. Key genes were identified through protein-protein interaction (PPI) network and KEGG pathway enrichment analyses. These genes underwent survival analysis and validation using data from The Cancer Genome Atlas (TCGA) and the Chinese Glioma Genome Atlas (CGGA).

**Results:**

Among 23 key genes, *CXCL8/IL8* and THBS1 were validated in TCGA and CGGA datasets. CXCL8 significantly correlated with progression-free survival (PFS) in Gr. 4 gliomas (*p* = 0.028) but not Gr. 3 (*p* = 0.522). ROC analysis confirmed its diagnostic accuracy (AUC: 0.899 in TCGA, 0.644 in CGGA). Immune infiltration analysis linked CXCL8 to macrophages (*p* < 0.001) and neutrophils (*p* < 0.001), particularly M2 macrophages.

**Conclusions:**

*CXCL8* has a significant impact on disease prognosis and tumor immunity in Gr. 4 gliomas. The association of *CXCL8* with M2 macrophage infiltration suggests that *CXCL8*-related markers could serve as potential prognostic biomarkers for differentiating between Gr. 4 and Gr. 3 gliomas.

**Supplementary Information:**

The online version contains supplementary material available at 10.1007/s12672-025-03982-2.

## Background

In recent decades, cancer treatment strategies have evolved from traditional surgery and chemotherapy to precision medicine approaches involving targeted therapies and immunotherapies. This transition is well-documented in recent reviews such as the comprehensive analysis by the U.S. National Cancer Institute, which outlines the historical progress and future directions of cancer therapies [1].

The histological grading of brain gliomas, as outlined in the World Health Organization (WHO) classification guidelines for central nervous system (CNS) tumors [2], has been a standard for over three decades. Pathologists are required to determine the grade of glioma based on its histological characteristics. Grade 4 (Gr. 4) gliomas are characterized by features such as nuclear atypia, hypercellularity, necrosis, and microvascular proliferation, while grade 3 (Gr. 3) gliomas display less severe histopathological traits. However, subjective grading by pathologists can be complex and challenging. Although molecular features are increasingly being used in glioma classification, histological grading remains a crucial step in the final diagnosis, significantly impacting clinical treatment decisions.

Adult-type diffuse Gr. 3 and Gr. 4 gliomas were reclassified into five subgroups in the 2021 edition [3]. A notable change is that the Gr. 4 glioblastoma category from the 2016 edition [4], which included IDH mutations with CDKN2A/B homozygous deletion, was revised to Gr. 4 astrocytoma in the 2021 edition [5]. Several studies have identified favorable molecular prognostic factors [6], such as IDH1/2 mutations [7, 8], 1p/19q codeletion [7], ATRX loss [9], TP53 wild type, TERT promoter wild type [7], and O^6^-methylguanine-DNA-methyltransferase (MGMT) promoter hypermethylation [8]. These studies indicate that the revised 2021 WHO classification of gliomas primarily focuses on disease prognosis. Therefore, identifying prognostic biomarkers that can distinguish between Gr. 3 and Gr. 4 gliomas is valuable for the clinical management of these conditions.

Histopathological classification and CNS WHO grading remain essential for providing an integrated diagnosis alongside molecular status. A molecular analysis report from the European Organization for Research and Treatment of Cancer (EORTC) 26,951 study [10] suggests that patients with Gr. 3 gliomas who have IDH mutations and 1p/19q codeletion, as well as other Gr. 3 glioma patients, should receive radiotherapy (RT) along with adjuvant chemotherapy using PVC (procarbazine, lomustine, and vincristine) or temozolomide (TMZ) [10, 11]. It is widely accepted that the prognosis for Gr. 3 glioma is generally better than that for Gr. 4 glioma, including astrocytoma [12]. Given the Significant differences in clinical outcomes and treatment guidelines between these two grades, there is an urgent need for objective biomarkers that can assist in accurate glioma grading and prognosis.

In this context, our study aims to identify robust molecular markers that can improve current histological grading practices and enhance prognostic stratification in gliomas.

To achieve this, we first analyzed differentially expressed genes (DEGs) between Gr. 3 and Gr. 4 gliomas in adults using datasets from the Gene Expression Omnibus (GEO). We then identified and validated key prognostic hub genes related to high-grade gliomas (HGG) using an external validation set. Finally, we explored the relationship between these prognostic gene markers and immune function to further differentiate between Gr. 3 and Gr. 4 gliomas.

## Materials and methods

### Microarray data from the gene expression omnibus (GEO)

We obtained two datasets, GSE4290 and GSE109857, from the GEO database (https://www.ncbi.nlm.nih.gov/geo) [13], both of which contain human whole-genome gene expression profiling data from glioma samples. The GSE4290 dataset [14], generated using the Affymetrix Human Genome U133 Plus 2.0 Array (GPL570), includes 31 Gr. 3 glioma samples and 77 glioblastoma Gr. 4 samples. The GSE109857 dataset [15], based on the Agilent-014850 Whole Human Genome Microarray 4 × 44 K G4112F (GPL6480), comprises 34 Gr. 3 glioma samples and 89 glioblastoma Gr. 4 samples (Supplementary Table S1).

Published on April 10, 2006, the GSE4290 dataset is classified solely by pathological grading. In contrast, GSE109857, published on Jul 15, 2020, offers additional genomic classification information. By integrating both datasets, our analysis of Gr. 4 gliomas is strengthened, particularly through the identification of prognostic genes among the DEGs. This strategy allows us to highlight staining markers that could be crucial in the pathological grading system.

To ensure consistency between platforms, we first converted each dataset’s probe-level data into gene-level matrices by averaging expression values of multiple probes targeting the same gene symbol. This preprocessing step reduced probe redundancy and allowed for fairer comparisons across platforms.

### Identification of differentially expressed genes (DEGs)

We used the GEO2R [16], an interactive online tool, to perform the analysis of DEGs by comparing whole-genome gene expression profiling data between Gr. 4 and Gr. 3 samples in the GSE4290 and GSE109857 datasets, respectively.

Because these two datasets are based on different microarray platforms (Affymetrix for GSE4290 and Agilent for GSE109857), direct merging could introduce batch effects and platform-related biases. Therefore, we performed differential expression analysis separately for each dataset and focused only on overlapping DEGs that appeared in both, enhancing the robustness and reproducibility of our findings across studies.

In GEO2R, R language was employed for data queries via the GEOquery package, while gene expression analysis was performed using the limma packages. The t-test was used to compare gene expression levels and calculate p-values, while the false discovery rate (FDR) was controlled for multiple tests using the Benjamini-Hochberg method [17]. The criteria for significance were set at an adjusted p-value < 0.05 and |log[fold change (FC)]| ≥ 1. The volcano plot was generated using the ggplot2 package in R (3.6.3).

### Protein-protein interaction (PPI) and KEGG pathway enrichment analyses

For the protein-protein interaction (PPI) network, the STRING database (https://string-db.org/) was used to obtain PPI pairs with a confidence score of 0.4 (medium confidence), which were then imported into Cytoscape software with the CytoNCA plugin to construct the PPI network. The key node (hub gene) was identified based on their degree score, which reflects the number of connections (edges) to a node, indicating its importance within the network. KEGG pathway enrichment analysis of these hub genes was conducted using the online tool DAVID (https://david.ncifcrf.gov/tools.jsp), with significance thresholds set at *p*-value < 0.05 and FDR < 0.25.

### Survival analysis and validation

Survival analysis was conducted using the low-grade glioma (LGG) and glioblastoma (GBM) datasets obtained from The Cancer Genome Altas (TCGA) (https://portal.gdc.cancer.gov/) [18, 19], Kaplan-Meier survival curves were generated, and the log-rank test was performed using the survival and survminer packages in R.

To validate the prognostic value of the candidate genes, we further used the mRNAseq_325 and mRNAseq_693 datasets, which include RNA-seq data and clinical information, from the Chinese Glioma Genome Atlas (CGGA) (https://www.cgga.org.cn/) [20]. Statistical analyses were performed using the Mann-Whitney U test in R 3.6.3 and results were visualized with the ggplot2 package.

### Gene set enrichment analysis (GSEA)

The R package DESeq2 was used with default settings to analyze *CXCL8* differential expression in TCGA RNA-seq data [21]. To account for multiple comparisons, the FDR was adjusted using the Benjamini‒Hochberg method. Following this, pathway analysis was performed using Gene Set Enrichment Analysis (GSEA) with curated gene sets (h.all.v7.2.symbols.gmt) via the R package clusterProfiler [22]. The results were then visualized using the ggplot2 graphics package.

### Gene expression and receiver operating characteristic (ROC) curve analysis

For the selected genes from the previous analysis, we conducted gene expression analysis using TCGA data. The nonparametric Mann-Whitney U test was applied to evaluate the differences in gene expression between Gr. 3 and Gr. 4 samples within the GBM-LGG cohort. To investigate the association between clinicopathological characteristics and *CXCL8* expression, samples were categorized into low and high-expression groups based on the median *CXCL8* expression level. The Chi-square test, implemented via the stats package, was used to assess these associations, with statistical significance set at p-value < 0.05. ROC curve analysis was then performed using the pROC package, and the distribution of gene expression as well as the ROC plots were visualized using the ggplot2 package.

### IL8/CXCL8 immunohistochemistry (IHC) staining

We collected samples from 12 high-grade glioma patients, including 4 with Gr. 3 gliomas and 8 with Gr. 4 gliomas, classified based on histological characteristics, and performed *CXCL8* immunohistochemistry (IHC) staining. *CXCL8* staining was performed using a mouse monoclonal antibody (clone 6217) raised against E. coli-derived recombinant human *IL8/CXCL8* (Ser28-Ser99, Accession # P10145), which consists of 99 amino acids from the human *CXCL8* protein (MAB208, R&D Systems). Before staining, formalin-fixed glioma tissue sections were deparaffinized with xylene and rehydrated in ethanol. The sections were then immersed in a phosphate-buffered saline (PBS) bath for 5 min and excess buffer was removed before proceeding.

The primary antibody was diluted 1:150 and incubated with the tissue sections for over 60 min. After incubation, the sections were washed twice with PBS for 5 min each. Secondary antibody incubation was conducted using an anti-mouse horseradish peroxidase (HRP) conjugate, followed by diaminobenzidine (DAB) chromogen staining for 20 min. The slides were then rinsed with distilled water and counterstained with Harris hematoxylin. As a negative control, some slides were processed without the primary antibody.

The staining intensity was assessed and categorized as follows: negative (< 10% positive cells), mild (10%-35% positive cells), moderate (35%-65% positive cells), and strong (> 65% positive cells). These evaluations were validated using an image analysis algorithm. All IHC images were confirmed to be derived from tumor regions by experienced pathologists to ensure interpretive accuracy.

### Tumor immune infiltration and immune cell marker correlation analysis

Finally, we utilized the single-sample gene set enrichment analysis (ssGSEA) algorithm to examine the infiltration levels of 24 immune cell subtypes in Gr. 4 gliomas to determine whether *CXCL8* expression is associated with immune infiltration. The enrichment levels of 24 tumor-infiltrating lymphocyte populations were assessed using the “GSVA” package, based on the ssGSEA method [23]. Additionally, we investigated the relationship between *CXCL8* expression and various immune cell gene markers using TIMER (https://cistrome.shinyapps.io/timer/), an interactive web interface designed for comprehensive analysis of tumor-immune interactions.

## Results

### Identification of DEGs between Gr. 3 and Gr. 4 gliomas from the GEO dataset

**Fig. 1:**
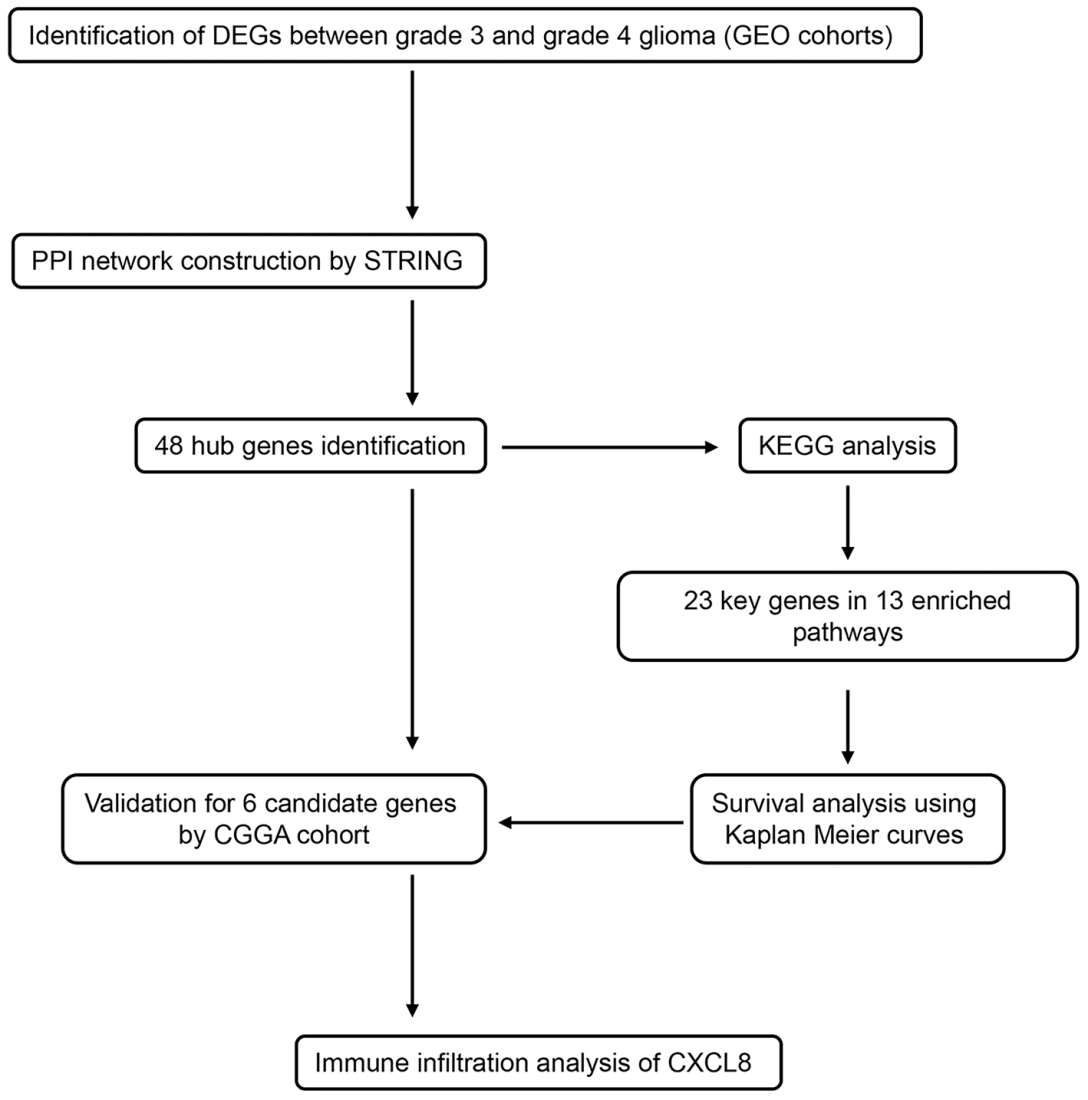
Flow chart of the study design

**Fig. 2:**
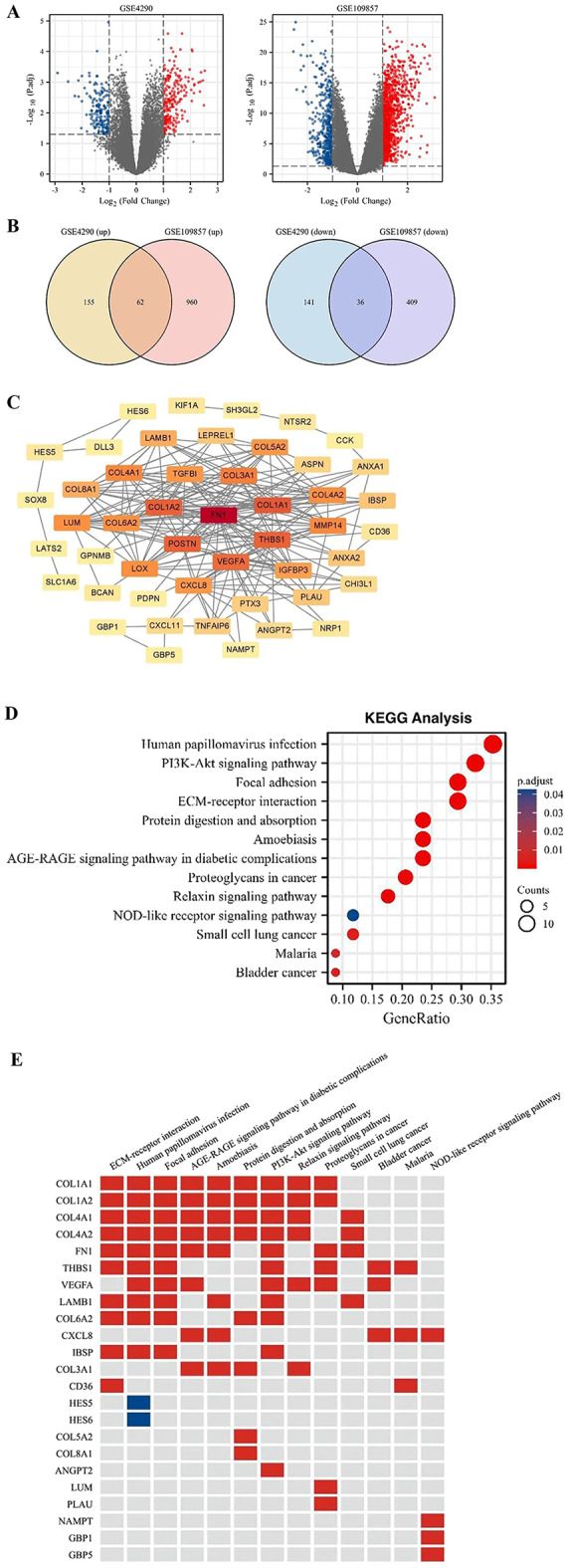
Identification of DEGs between Gr. 3 and Gr. 4 gliomas in two GEO datasets. **A** Volcano plot highlighting significant DEGs between Gr. 3 and Gr. 4 gliomas. **B** Venn diagram showing the overlap of upregulated DEGs (left) and downregulated DEGs (right) between the two datasets. **C** Extraction of 48 hub genes (degree ≥ 2) from the PPI network of common DEGs across both datasets. **D** Bubble plot depicting 13 enriched pathways identified from KEGG pathway enrichment analysis of 48 hub genes (p-value < 0.05). The color gradient reflects the adjusted p-values of KEGG pathway enrichment. **E** Identification of 23 key genes involved in the enriched pathways

The study’s workflow is illustrated in Fig. 1. Differentially expressed genes (DEGs) were identified using gene expression profiles from the GSE4290 and GSE109857 datasets. In the GSE4290 dataset, 217 genes were upregulated, and 177 were downregulated when comparing gliomas of histologic Gr. 4 and 3. Similarly, the GSE109857 dataset revealed 1,022 upregulated genes and 445 downregulated genes. The corresponding volcano plots are presented in Fig. 2A. As shown in the Venn diagrams in Fig. 2B, a total of 98 overlapping DEGs were identified between the two databases, including 62 upregulated and 36 downregulated genes.

Figure 2C shows the protein-protein interaction networks (PPI) network. A total of 97 nodes and 232 edges were identified from the PPI network. Using the CytoNCA plugin, 48 DEGs were identified as hub genes according to degree value ≥ 2. These hub genes were then subjected to KEGG pathway enrichment analysis using the DAVID online tool. The significantly enriched pathways were Human papillomavirus infection, PI3K-Akt signaling pathway, Focal adhesion, ECM-receptor interaction, Protein digestion and absorption, Amoebiasis, AGE-RAGE signaling pathway in diabetic complications, Proteoglycans in cancer, Relaxin signaling pathway, NOD-like receptor signaling pathway, Small cell lung cancer, Malaria, and Bladder cancer (Fig. 2D). Some enriched pathways may seem unrelated to glioma but likely reflect the presence of shared immune-related genes commonly deregulated in tumors. To refine the list of 48 hub genes and identify those that distinguish Gr. 3 and Gr. 4 gliomas, 23 genes (21 upregulated and 2 downregulated) involved in the significant pathways were identified as “key genes” (Fig. 2E). These key genes were further validated through survival analysis to assess their clinical relevance.

### Prognostic genes identification and validation using survival analysis

To assess the prognostic value of these 23 key genes, including their impact on overall survival (OS) and progression-free survival (PFS) in Gr. 3 and Gr. 4 gliomas patients, we conducted Kaplan-Meier (K-M) analysis using the RNA-seq data and clinical information (LGG and GBM) obtained from the TCGA database (Supplementary Table S2).

In the survival analysis conducted for Gr. 4 glioma patients, six genes that were significantly associated with prognosis were defined as candidate prognostic genes. The expression levels of *THBS1* (HR = 1.40, *P* = 0.046), *CXCL8* (HR = 1.45, *P* = 0.028), *IBSP* (HR = 1.52, *P* = 0.012), *COL8A1* (HR = 1.15, *P* = 0.023) and *GBP5* (HR = 1.43, *P* = 0.035) were associated with a shorter progression-free interval, while the expression level of *HES5* (HR = 0.69, *P* = 0.027) was significantly associated with better overall survival in these patients.

We also evaluated the prognostic significance of these six genes in Gr. 3 glioma patients. Among them, four genes, including *THBS1* (HR = 1.51, *P* = 0.035), *IBSP* (HR = 2.18, *P* < 0.001), *COL8A1* (HR = 2.66, *P* < 0.001) and *GBP5* (HR = 2.43, *P* < 0.001), were significantly associated with poor progression-free survival. Notably, higher expression of *HES5* (HR = 0.47, *P* < 0.001) was associated with better prognosis, suggesting a protective role of *HES5* in Gr. 3 gliomas as well. Additionally, three genes, including *IBSP* (HR = 1.86, *P* = 0.005), *COL8A1* (HR = 2.70, *P* < 0.001), and *GBP5* (HR = 2.09, *P* = 0.001), were significantly associated with poor overall survival. Again, higher *HES5* (HR = 0.54, *P* = 0.004) expression was linked to improved survival, consistent with its protective effect. (Table 1 and Supplementary Table S2). To validate these results, another two datasets (mRNAseq_325 and mRNAseq_693) from the CGGA were also utilized. Two genes, *THBS1* and *CXCL8*, remained significantly associated with poor overall survival in Gr. 4 but not in Gr. 3 patients (Table 2).

**Table 1:** The six genes significantly associated with OS and PFS in patients with grade 3 and 4 glioma using data from TCGA

Gene	TCGA							
	Grade 3				Grade 4			
	OS		PFS		OS		PFS	
	HR (95% CI)	P value	HR (95% CI)	P value	HR (95% CI)	P value	HR (95% CI)	P value
*THBS1*	1.15 (0.74–1.78)	0.536	1.51 (1.03–2.21)	0.035^*^	1.19 (0.84–1.67)	0.312	1.40 (0.99–1.97)	0.046^*^
*CXCL8*	0.95 (0.61–1.47)	0.801	0.88 (0.60–1.30)	0.522	1.35 (0.96–1.89)	0.077	1.45 (1.03–2.03)	0.028^*^
*IBSP*	1.86 (1.20–2.87)	0.005^*^	2.18 (1.48–3.20)	< 0.001^*^	1.29 (0.92–1.81)	0.131	1.52 (1.08–2.14)	0.012^*^
*HES5*	0.54 (0.34–0.84)	0.004^*^	0.47 (0.32–0.69)	< 0.001^*^	0.69 (0.49–0.97)	0.027^*^	0.74 (0.53–1.04)	0.072
*COL8A1*	2.70 (1.74–4.20)	< 0.001^*^	2.66 (1.80–3.91)	< 0.001^*^	1.15 (0.82–1.61)	0.407	1.47 (1.04–2.06)	0.023^*^
*GBP5*	2.09 (1.35–3.23)	0.001^*^	2.43 (1.65–3.58)	< 0.001^*^	1.21 (0.87–1.70)	0.247	1.43 (1.02-2.00)	0.035^*^

*OS* overall survival,* PFS* progression-free survival,* HR* hazard ratio,* CI* confidence interval

**Table 2:** Validation of associations between the six genes and OS in patients with grade 3 and 4 gliomas using data from CGGA

Gene	CGGA			
	Grade 3		Grade 4	
	HR (95% CI)	P value	HR (95% CI)	P value
*THBS1*	1.22 (0.72–2.09)	0.456	1.76 (1.23–2.52)	0.001^*^
*CXCL8*	1.35 (0.79–2.30)	0.271	1.61 (1.12–2.30)	0.006^*^
*IBSP*	1.76 (1.03–3.01)	0.036^*^	1.37 (0.96–1.95)	0.075
*HES5*	0.85 (0.50–1.46)	0.563	1.05 (0.74–1.50)	0.767
*COL8A1*	2.60 (1.50–4.50)	0.085	1.40 (0.98–1.99)	0.059
*GBP5*	1.82 (1.06–3.12)	0.025^*^	1.32 (0.93–1.88)	0.113

*OS* overall survival,* HR* hazard ratio,* CI* confidence interval

**Fig. 3:**
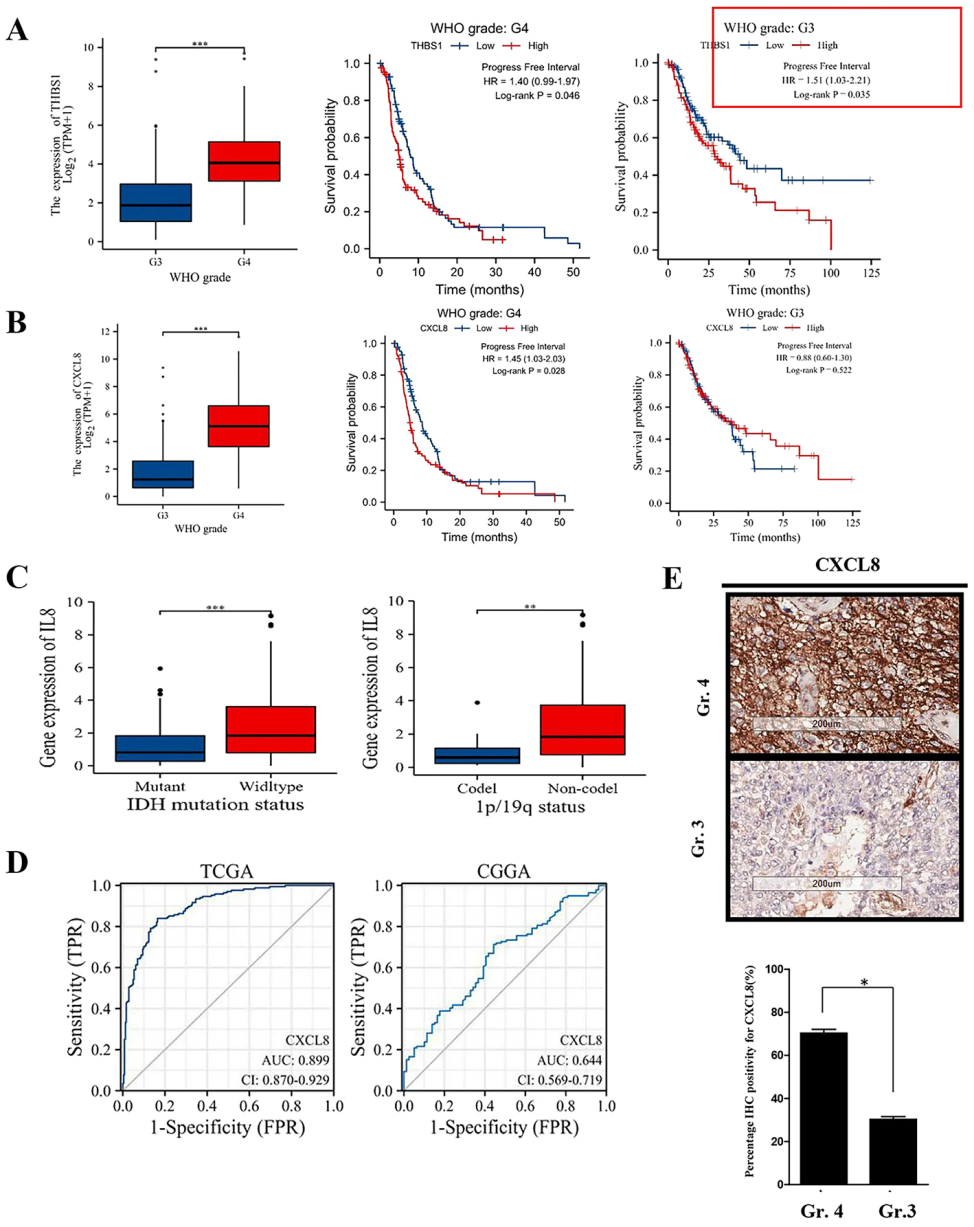
Identification of prognostic genes and analysis of mRNA expression in Gr. 3 and Gr. 4 gliomas using TCGA data. **A** Gene expression and Kaplan-Meier survival analysis for *THBS1*. **B** Gene expression and Kaplan-Meier survival analysis for *CXCL8*. **C** The distribution of *CXCL8/IL8* expression level on the IDH status (left, wildtype: Gr. 4 glioblastoma vs. Mutant: IDH mutant Gr. 4 glioma) and 1p19q codeletion status (right, Non-codel: non-codeletion, IDH-mutant Gr. 4 glioma vs. Codel: codeletion, IDH-mutant Gr. 3 glioma). **D** Receiver operating characteristic (ROC) curve analysis for discriminating Gr. 4 gliomas based on *CXCL8* expression in TCGA (left) and CGGA (right) datasets. **E** Representative examples of *CXCL8* IHC staining (upper), with a bar graph showing the average IHC positivity between Gr. 3 and Gr. 4 gliomas for *CXCL8* (lower, *: p-value < 0.05)

The expression levels of *THBS1* and *CXCL8* in Gr. 3 and Gr. 4 gliomas are shown in Fig. 3A and B, along with survival curves for these genes using TCGA data. Validation with CGGA data (Supplementary Figure S1) confirmed these results, suggesting that *THBS1* and *CXCL8* may play critical roles in distinguishing between Gr. 4 and Gr. 3 gliomas.

### CXCL8 as a unique prognostic gene for predicting grade 4 glioma outcomes

Given the potentially significant impact of *CXCL8* on the prognosis of Gr. 4 glioma patients, we performed the GSEA analysis on high and low expression levels of *CXCL8*, categorized based on the median expression value using TCGA GBM RNA-seq data. The high-expression group for *CXCL8* showed significant enrichment in pathways including *ANGIOGENESIS*,* INFLAMMATORY RESPONSE*, *TNFA SIGNALING VIA NFKB*, *IL6 JAK STAT3 SIGNALING*, and *ALLOGRAFT REJECTION* (Supplementary Figure S2).

Additionally, we examined the relationship between *CXCL8* expression levels and glioma grade, as well as other clinical factors using the TCGA dataset. As summarized in Table 3, *CXCL8* mRNA expression was associated with WHO grade (Gr. 4), IDH status (wild-type), and 1p/19q status (non-codel).

**Table 3:** Association of *CXCL8* expression levels with clinicopathological characteristics of patients with glioma

Characteristics	Low expression of CXCL8 *N* = 302	High expression of CXCL8 *N* = 319	*P* value
*WHO grade*,* (%)*			< 0.001
Gr. 2	148 (23.8%)	74 (11.9%)	
Gr.3	146 (23.5%)	99 (15.5%)	
Gr.4	8 (1.3%)	146 (23.5%)	
*IDH status*,* n (%)*			< 0.001
Wildtype	53 (8.5%)	181 (29.1%)	
Mutant	249 (40.1%)	138 (22.2%)	
*1p/19q codeletion*,* n (%)*			< 0.001
Non-codel	234 (32%)	268 (43.2%)	
Codel	103 (16.6%)	51 (8.2%)	

*Gr.* grade

*CXCL8* (C-X-C motif chemokine ligand 8) is also named interleukin 8 (*IL8*). The classification of WHO gliomas is also based in part on the IDH mutation status and 1p/19q codeletion; thus, we next analyzed the association of *CXCL8* with these markers in Gr. 3 and Gr. 4 gliomas, respectively. The results showed that *CXCL8* was expressed in IDH wt. Gr. 4 glioblastoma at higher levels than in IDH-mutant, Gr. 4 glioma with IDH mutation (Fig. 3C, left). In contrast, a significant finding was that *CXCL8* expression in 1p/19q non-codeletion, IDH-mutant Gr. 4 glioma is higher than that in the IDH-mutant, Gr. 3 glioma codeletion group (Fig. 3C, right). Unfortunately, the CDKN2A/B homozygous deletion status was not related to *CXCL8* in the TCGA dataset.

To assess the unique role of *CXCL8* in distinguishing Gr. 4 gliomas from Gr. 3 gliomas, we performed ROC curve analysis using TCGA and CGGA datasets. The area under the curve (AUC) was 0.899 for differentiating Gr. 4 from Gr. 3 gliomas in the TCGA cohort and 0.644 in the CGGA dataset (Fig. 3D), indicating that *CXCL8* plays a significant role in distinguishing between these grades of glioma.

Additionally, we collected samples from 12 high-grade glioma patients, including 4 with Gr. 3 gliomas and 8 with Gr. 4 gliomas, based on histological characteristics, and performed *CXCL8* immunohistochemistry (IHC) staining. The results showed more pronounced *CXCL8* expression in Gr. 4 gliomas compared to Gr. 3 gliomas (Fig. 3E, upper). The average positive percentage of *CXCL8* staining was significantly higher in Gr. 4 gliomas than in Gr. 3 gliomas (Fig. 3E, lower). These results further confirm the potential of *CXCL8* as a predictive marker for differentiating glioma grades in clinical settings.

### CXCL8 correlates with immune infiltration and M2 macrophage polarization, especially in recurrent grade 4 gliomas

**Fig. 4:**
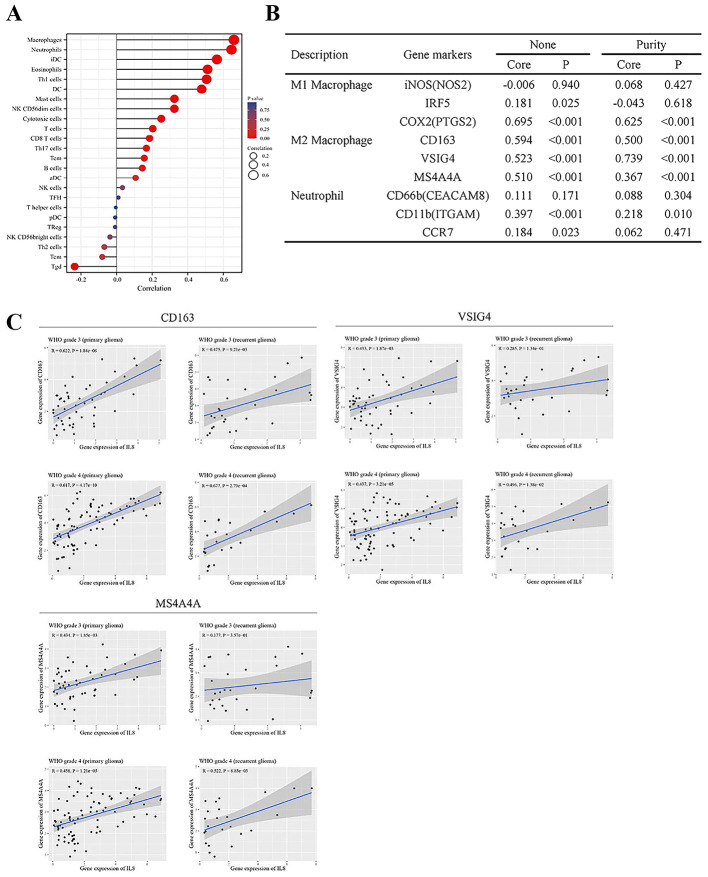
Associations between *CXCL8* expression and the tumor microenvironment. **A** Correlation analysis between *CXCL8*/*IL8* expression and immune cell infiltration in Gr. 4 glioma using the ssGSEA algorithm, based on TCGA database. The color gradient reflects the adjusted p-values of pathway enrichment. **B** The correlation between *CXCL8* expression and marker genes of various immune cells was analyzed using the TIMER database. **C** Scatter plots depicting the relationship between *CXCL8* expression and the expression of M2 macrophage marker genes (CD163, VSIG4, and MS4A4) in the CGGA database

To understand the potential role of *CXCL8* in shaping the immune microenvironment of high-grade gliomas, we investigated its association with various immune cell populations, aiming to elucidate possible mechanisms by which *CXCL8* contributes to glioma progression. *CXCL8* is a chemokine secreted by multiple immune and non-immune cells, including macrophages, neutrophils, T lymphocytes, fibroblasts, and epithelial cells. Using the ssGSEA algorithm, we explored the infiltration levels of 24 immune cell subtypes in Gr. 4 gliomas to observe whether *CXCL8* expression is associated with immune infiltration. The main immune cells affected by *CXCL8* expression were macrophages, neutrophils, immature DCs (iDCs), eosinophils, Th1 cells, DCs, mast cells, NK CD56dim cells, cytotoxic cells, T cells, CD8 T cells, Th17 cells, T effector memory (Tem) cells and T gamma delta (Tgd). Among them, the levels of macrophages (*r* = 0.660, *P* < 0.001) and neutrophils (*r* = 0.637, *P* < 0.001) showed the strongest positive correlations with *CXCL8* expression (Fig. 4A), suggesting their prominent role in the *CXCL8*-associated immune landscape.

To further delineate the subtype-specific association, we evaluated the correlation between *CXCL8* and canonical immune cell markers using the TIMER database [24]. After adjusting for tumor purity, *CXCL8* expression showed strong positive correlations with M2 macrophage markers (CD163, VSIG4, MS4A4A) in Gr. 4 gliomas (Fig. 4B), but not with M1 markers. These findings imply a potential immunosuppressive role of *CXCL8* through M2 macrophage polarization.

We further validated these associations using the CGGA database across both primary and recurrent HGG samples. While *CXCL8* remained significantly correlated with M2 markers in both Gr. 3 and Gr. 4 gliomas, the correlation markedly strengthened in recurrent Grade 4 gliomas. Specifically, the R-values for CD163, VSIG4, and MS4A4A increased from 0.617 to 0.677, 0.437 to 0.496, and 0.458 to 0.522, respectively. In contrast, correlations decreased in recurrent Gr. 3 gliomas. These patterns suggest that *CXCL8*-associated M2 macrophage infiltration may be particularly relevant to tumor progression and recurrence in Grade 4 gliomas (Fig. 4C).

Together, these results highlight the key role of *CXCL8* in shaping an immunosuppressive microenvironment in Grade 4 gliomas through its strong association with M2 macrophage-related functions. This strengthens its potential clinical utility as a target for immune-modulating strategies in high-grade gliomas.

## Discussion

In this study, we first analyzed the DEGs between Gr. 3 and Gr. 4 gliomas in adults using GEO datasets, identifying and validating 23 key prognostic hub genes associated with HGG. The predominance of upregulated genes observed in our analysis may reflect the activation of proliferative and invasive programs during glioma progression. Such transitions are often accompanied by phenotypic shifts in tumor subtypes and immunological remodeling of the tumor microenvironment [25]. In particular, several collagen-related genes (e.g., *COL1A1*,* COL3A1*,* COL5A1*) were significantly upregulated, suggesting increased extracellular matrix deposition and the establishment of an immunosuppressive microenvironment in Grade 4 gliomas. Recent studies have also demonstrated that collagen gene expression is closely associated with epithelial–mesenchymal transition (EMT) and immune cell infiltration in gliomas [26].

We then conducted survival analysis on sample sets from the TCGA and CGGA databases, confirming the prognostic significance of two genes, *THBS1* and *CXCL8*. These genes proved effective in distinguishing IDH wild-type glioblastoma as Gr. 4 glioma and 1p/19q co-deleted gliomas as Gr. 3 glioma. Additionally, we explored the relationship between these prognostic genes and immune functions, offering further insights into differentiating between Gr. 3 and Gr. 4 gliomas.

Among the identified genes, thrombospondin-1 (*THBS1*) emerged as a significant player in glioma pathology. *THBS1* is a secreted protein involved in various cellular processes, including cell adhesion, angiogenesis, apoptosis, and immune response [27]. *THBS1* has been recognized as a key biomarker for predicting the mesenchymal subtype of glioblastoma and aiding in histopathological diagnosis. Importantly, overexpression of *THBS1* has been associated with a poor prognosis in Gr. 4 gliomas. Moreover, *THBS1* is linked to inflammatory and immune responses in glioblastoma, reinforcing its role as a critical factor in tumor progression [28]. Through our study and validation with the CGGA Kaplan-Meier analysis, *THBS1* was confirmed as a central prognostic gene in Gr. 4 gliomas, consistent with previous research.

In addition to *THBS1*, our study also highlighted the importance of interleukin-8 (*IL8*, *CXCL8*) in glioma prognosis. Previous studies have shown a correlation between *CXCL8* expression and poor outcomes in various cancers, including glioblastoma [29]. Our findings suggest that *CXCL8* plays a critical role in distinguishing between Gr. 3 and Gr. 4 gliomas. Notably, *CXCL8* expression has been linked to higher plasma levels in glioblastoma patients, which correlates with shorter overall survival (OS) [30]. Our data also indicate that *CXCL8* is a significant prognostic biomarker associated with poor progression-free survival (PFS) in glioblastoma patients from the TCGA dataset and is a predictor of poor OS in the CGGA cohort. Despite *CXCL8’s* strong discriminatory performance in the TCGA dataset (AUC = 0.899), its AUC in the CGGA cohort was more modest (AUC = 0.644). This discrepancy may reflect population-specific differences, as well as variations in sequencing platforms and clinical data quality between datasets. Nevertheless, its consistent statistical significance across both cohorts supports the robustness of *CXCL8* as a potential biomarker. The observed difference in performance also highlights the importance of investigating population-dependent regulatory mechanisms in future studies.

While *CXCL8* has been previously implicated in glioma progression and immune infiltration, our study provides new insights by demonstrating that *CXCL8* expression exhibits grade‑specific prognostic value, significant in Gr. 4 gliomas but not in Gr. 3. This distinction aligns with the notion that tumor-associated macrophages (TAMs), including M2-like subtypes, are significantly enriched in high‑grade gliomas and correlate with poor prognosis [31]. Such grade-dependent roles of *CXCL8* have remained under-characterized in prior studies. These findings suggest that *CXCL8* may serve as a grade-specific biomarker, contributing to both prognostic assessment in high-grade gliomas and the stratification of glioma subtypes with distinct immune landscapes. This grade-specific effect may be due to the distinct tumor microenvironment of Gr. 4 gliomas, which are often IDH-wildtype and enriched with immunosuppressive M2 macrophages, allowing *CXCL8* to exert greater biological influence compared to Gr. 3 gliomas.

Furthermore, we observed a notable increase in the correlation between *CXCL8* expression and M2 macrophage infiltration in recurrent glioblastoma, a finding not extensively documented previously. Recurrent GBMs are known to have an altered tumor immune microenvironment driven by TAM-mediated immunosuppression [32]. Our data suggest that *CXCL8* may contribute more prominently to tumor progression or immune evasion mechanisms during recurrence, possibly via modulation of macrophage polarization or cytokine signaling pathways.

Although our current study does not provide direct mechanistic experiments, the observed grade-dependent expression and immune associations support a hypothesis that *CXCL8* may act as a driver of immunosuppressive remodeling in the Gr. 4 glioma microenvironment, with implications for recurrence and resistance. This concept is consistent with emerging evidence highlighting *CXCL8*’s role in reprogramming the glioblastoma stem‑cell niche toward a mesenchymal, TAM-polarizing state [33]. Similar TCGA-based transcriptomic approaches have been applied in other solid tumors to identify immune-related prognostic biomarkers, supporting the validity of our analytic strategy [34, 35]. Further experimental investigations, including functional assays and co-localization studies, are warranted to validate this mechanistic link and explore its therapeutic implications.

Temozolomide (TMZ), a common alkylating agent used in adjuvant chemotherapy for HGG, has been shown to upregulate the *CXCL8*-dependent CXCL2/CXCR2 axis, promoting angiogenesis and the recruitment of tumor-associated macrophages. Combination therapy targeting CXCR2 in conjunction with TMZ has been suggested to overcome therapeutic resistance induced by TMZ [36]. Furthermore, HGGs are known to be resistant to immune checkpoint blockade therapy, partly due to a subpopulation of CD4 + T cells that express *CXCL8* and contribute to tumor growth and reduced efficacy of immune checkpoint inhibitors. Blocking *CXCL8* or its receptors, *CXCR1/2* has been shown to enhance anti-PD-1-mediated antitumor immunity, making *CXCL8* a potential target for combination immunotherapy in gliomas [37]. Future studies integrating transcriptomic data from TMZ-treated glioma samples may help clarify whether *CXCL8* expression influences drug response, thereby enhancing its clinical relevance.

The differential prognosis of patients with Gr. 3 and Gr. 4 gliomas, even after adjuvant therapy, underscores the need for more effective therapeutic strategies. Our study suggests that *CXCL8*expression, as determined by immunohistochemistry (IHC) staining, could be used to stratify glioma patients and predict their response to TMZ treatment. Targeting *CXCL8* in combination with other treatments may improve therapeutic outcomes in gliomas with high *CXCL8* expression, offering a promising approach for treating HGG. In addition, although direct evidence is limited, some studies have reported a correlation between tumor tissue and circulating *CXCL8* levels in glioma patients, suggesting the potential of serum or CSF *CXCL8* as a non-invasive biomarker [38]. This highlights the potential of incorporating *CXCL8* into liquid biopsy strategies, such as using circulating cytokine levels to monitor tumor progression or immune modulation in a minimally invasive manner.

Our IHC analysis was limited to 12 samples (including only 4 Gr. 3 gliomas), due to material availability and ethical constraints. Despite the small sample size, the results were consistent with transcriptomic findings, suggesting robustness. The IHC findings provide preliminary and supportive evidence that warrants further validation in larger, prospectively collected cohorts.

Recent advancements in machine learning systems for gliomas have also highlighted the role of macrophage recruitment and M2 polarization in connecting metabolic patterns with tumor-infiltrating immune cells (TIICs) in malignant gliomas [39]. TIIC-related long non-coding RNA (lncRNA) signatures have been established as indicators of immune cell infiltration and predictors of immunotherapeutic response in Gr. 3 and Gr. 4 gliomas. *CXCL8*, as a biomarker associated with M2 macrophage infiltration, could play a pivotal role in developing predictive systems for glioma treatment. Our study found that *CXCL8* expression was associated with increased levels of immune cells, including macrophages and neutrophils, in gliomas. Specifically, *CXCL8* expression was highly correlated with M2 macrophage infiltration in both primary and recurrent glioblastoma, further emphasizing its potential as a target for future therapeutic strategies. These findings reinforce the relevance of *CXCL8* in the immunosuppressive microenvironment of Gr. 4 gliomas, particularly through its association with M2 macrophage-related pathways, highlighting its potential as a clinically meaningful biomarker and therapeutic target. *CXCL8* immunostaining showed consistent and interpretable patterns. While no universally accepted cutoff value has been established, the consistent staining pattern observed in our study supports its potential as a practical and reproducible biomarker in clinical settings, warranting further validation in larger cohorts. In our IHC staining results, we observed that *CXCL8* signal was predominantly located along the cell periphery. Although *CXCL8* is a secreted cytokine, previous studies have demonstrated that it can remain associated with the cell membrane through interactions with glycosaminoglycans such as heparan sulfate [40]. This could explain the membrane-like distribution pattern we observed, which may have biological significance in recruiting immune cells and shaping an immunosuppressive tumor microenvironment. The precise cellular origin of *CXCL8* in the tumor microenvironment remains to be fully delineated, current evidence suggests that both tumor cells and infiltrating immune cells may contribute to its expression.

The primary aim of this study was to identify candidate biomarkers capable of distinguishing between glioma grades and to provide a foundation for future mechanistic research. Although this study did not delineate the exact cellular origin of *CXCL8* expression, our findings offer valuable direction for future investigations. Our study did not distinguish whether *CXCL8* was produced by tumor cells or immune infiltrates, which remains a limitation. Approaches such as single-cell transcriptomics or multiplex immunostaining will be instrumental in clarifying whether *CXCL8* expression is tumor-intrinsic or derived from infiltrating immune cells, thereby enhancing our understanding of its prognostic and therapeutic relevance in a cell-type–specific context.

In addition to evaluating individual biomarker performance, future studies should also explore whether combined expression patterns of *CXCL8* and *THBS1* could improve discriminatory power between glioma grades. Multivariate biomarker integration within larger and clinically annotated cohorts may further support their utility in patient stratification and clinical decision-making. Additionally, integrating *CXCL8* with well-established prognostic markers such as MGMT promoter methylation or TERT mutations could enhance its clinical predictive value. *CXCL8* may also be incorporated into composite scoring systems alongside immune scores or molecular classifiers to further refine risk assessment and therapeutic decision-making. Due to dataset limitations, our survival analyses could not adjust for potential confounders such as IDH mutation, 1p/19q codeletion, age, and treatment, which may influence clinical outcomes. Future studies incorporating multivariate models with comprehensive clinical, molecular, and immunological annotations are warranted to validate the independent prognostic role of *CXCL8* and enhance its utility in glioma risk stratification.

Furthermore, to identify differentially expressed genes (DEGs) distinguishing Gr. 3 from Gr. 4 gliomas, we utilized two publicly available datasets: GSE4290 and GSE109857, which are based on Affymetrix and Agilent platforms respectively. Rather than merging these datasets which would introduce confounding batch effects due to differing experimental platforms and preprocessing pipelines, we performed differential expression analyses independently. For each dataset, probe-level data were collapsed into gene-level matrices by averaging the expression of probes mapping to the same gene symbol. This allowed consistent gene-level comparison and mitigated platform-specific probe redundancy. We then focused on the intersecting DEGs, representing genes consistently dysregulated across both studies. This approach, though conservative, increases the credibility and reproducibility of the selected genes by confirming their robustness in independent cohorts. Since we did not integrate the datasets, batch effect correction was not necessary in our workflow. Although the number of intersecting DEGs used for pathway enrichment analysis was limited, these genes were derived from two independent platforms, lending credibility to the findings. Despite the small number of enriched pathways, the results provided a meaningful direction for subsequent analyses.

## Conclusions

In this study, we identified *CXCL8* as a potential biomarker for classifying HGG. However, *CXCL8* exerts various effects on disease prognosis and tumor immunity. The infiltration of *CXCL8*-associated M2 macrophages may serve as a distinguishing factor between Gr. 3 and Gr. 4 gliomas. Future investigations, including immunohistochemical staining of HGG and survival analysis based on *CXCL8* expression, could offer valuable insights into its potential role in glioblastoma treatment.

## Supplementary Information

Below is the link to the electronic supplementary material.

Supplementary Material 1

Supplementary Material 2

## Data Availability

The datasets used and/or analyzed during the current study are available from the corresponding author on request.

## Abbreviations

PPI: Protein–protein interaction
TCGA: The Cancer Genome Atlas
CGGA: Chinese Glioma Genome Atlas
IHC: Immunohistochemistry (IHC)
OS: Overall survival
PFS: Progression-free survival
CNS: Central nervous system
Gr.: Grade
