# Supplementary material for: M2 macrophage infiltration associated with CXCL8 predicts grade 4 prognosis and differentiates glioma grades
Source: Discov Oncol. 2025 Dec 23;16:2215. doi: 10.1007/s12672-025-03982-2 (PMC12728105; doi:10.1007/s12672-025-03982-2)
Supplement: Supplementary file 1 — Supplementary Material 1 [file 12672_2025_3982_MOESM1_ESM.docx]

**Supplementary Table S1. Details of the two datasets from the GEO.**

| **Dataset** | **Grade 3** | **Grade 4** |
| --- | --- | --- |
| GSE4290 | 31 | 77 |
| GSE109857 | 34 | 89 |
| Total | 65 | 166 |

GEO, Gene Expression Omnibus.

**Supplementary Table S2**. **The relationships between 23 key gene levels, OS, and PFS in patients with grade 3 or 4 glioma.**

|  | **TCGA Grade 4** | | | | **TCGA Grade 3** | | | |
| --- | --- | --- | --- | --- | --- | --- | --- | --- |
| **Gene** | **OS** | | **PFS** | | **OS** | | **PFS** | |
|  | HR  (95% CI) | P value | HR  (95% CI) | P value | HR  (95% CI) | P value | HR  (95% CI) | P value |
| COL1A1 | 1.28 (0.91-1.81) | 0.137 | 1.32 (0.94-1.85) | 0.101 | 1.56 (1.01-2.42) | 0.039^*^ | 2.03 (1.38-2.97) | <0.001^*^ |
| COL1A2 | 1.24 (0.88-1.74) | 0.204 | 1.19 (0.85-1.67) | 0.292 | 1.63 (1.05-2.52) | 0.026^*^ | 1.90 (1.29-2.78) | 0.001^*^ |
| COL4A1 | 1.11 (0.79-1.56) | 0.529 | 1.15 (0.82-1.61) | 0.42 | 2.46 (1.59-3.81) | <0.001^*^ | 2.73 (1.86-4.03) | <0.001^*^ |
| COL4A2 | 1.14 (0.81-1.59) | 0.452 | 1.18 (0.84-1.65) | 0.34 | 2.30 (1.48-3.57) | <0.001^*^ | 2.65 (1.80-3.92) | <0.001^*^ |
| FN1 | 1.23 (0.87-1.73) | 0.221 | 1.21 (0.87-1.70) | 0.249 | 1.78 (1.15-2.76) | 0.01^*^ | 2.03 (1.39-2.98) | <0.001^*^ |
| THBS1 | 1.19 (0.84-1.67) | 0.312 | 1.40 (0.99-1.97) | 0.046^*^ | 1.15 (0.74-1.78) | 0.536 | 1.51 (1.03-2.21) | 0.035^*^ |
| VEGFA | 1.36 (0.97-1.91) | 0.068 | 1.19 (0.85-1.68) | 0.295 | 2.24 (1.43-3.50) | <0.001^*^ | 2.46 (1.65-3.66) | <0.001^*^ |
| LAMB1 | 1.30 (0.92-1.83) | 0.117 | 1.36 (0.97-1.91) | 0.065 | 1.50 (0.97-2.32) | 0.06 | 1.80 (1.23-2.65) | 0.002^*^ |
| COL6A2 | 1.38 (0.98-1.94) | 0.054 | 1.28 (0.91-1.80) | 0.139 | 1.48 (0.96-2.29) | 0.07 | 1.87 (1.27-2.74) | 0.001^*^ |
| CXCL8 | 1.35 (0.96-1.89) | 0.077 | 1.45 (1.03-2.03) | 0.028^*^ | 0.95 (0.61-1.47) | 0.801 | 0.88 (0.60-1.30) | 0.522 |
| IBSP | 1.29 (0.92-1.81) | 0.131 | 1.52 (1.08-2.14) | 0.012^*^ | 1.86 (1.20-2.87) | 0.005^*^ | 2.18 (1.48-3.20) | <0.001^*^ |
| COL3A1 | 1.12 (0.80-1.57) | 0.51 | 1.19 (0.85-1.67) | 0.296 | 1.61 (1.04-2.49) | 0.031^*^ | 1.95 (1.33-2.85) | 0.001^*^ |
| CD36 | 0.95 (0.68-1.33) | 0.751 | 1.00 (0.71-1.40) | 1 | 1.21 (0.78-1.87) | 0.381 | 1.21 (0.83-1.78) | 0.317 |
| HES5 | 0.69 (0.49-0.97) | 0.027^*^ | 0.74 (0.53-1.04) | 0.072 | 0.54 (0.34-0.84) | 0.004^*^ | 0.47 (0.32-0.69) | <0.001^*^ |
| HES6 | 0.84 (0.60-1.18) | 0.312 | 0.72 (0.51-1.01) | 0.05 | 0.51 (0.33-0.79) | 0.002^*^ | 0.47 (0.32-0.70) | <0.001^*^ |
| COL5A2 | 0.99 (0.71-1.39) | 0.964 | 1.24 (0.89-1.74) | 0.201 | 2.03 (1.31-3.14) | 0.002^*^ | 2.51 (1.71-3.68) | <0.001^*^ |
| COL8A1 | 1.15 (0.82-1.61) | 0.407 | 1.47 (1.04-2.06) | 0.023^*^ | 2.70 (1.74-4.20) | <0.001^*^ | 2.66 (1.80-3.91) | <0.001^*^ |
| ANGPT2 | 1.12 (0.80-1.57) | 0.495 | 1.12 (0.80-1.57) | 0.51 | 2.26 (1.46-3.50) | <0.001^*^ | 2.30 (1.57-3.39) | <0.001^*^ |
| LUM | 1.00 (0.71-1.40) | 0.996 | 1.23 (0.88-1.73) | 0.217 | 1.35 (0.87-2.09) | 0.172 | 1.71 (1.16-2.50) | 0.006^*^ |
| PLAU | 1.21 (0.86-1.70) | 0.26 | 1.34 (0.96-1.88) | 0.082 | 1.75 (1.13-2.71) | 0.011^*^ | 1.88 (1.28-2.75) | 0.001^*^ |
| NAMPT | 1.35 (0.96-1.89) | 0.077 | 1.36 (0.97-1.91) | 0.066 | 1.45 (0.94-2.25) | 0.082 | 2.04 (1.39-2.99) | <0.001^*^ |
| GBP1 | 0.89 (0.64-1.25) | 0.492 | 1.04 (0.75-1.46) | 0.798 | 2.49 (1.60-3.87) | <0.001^*^ | 2.18 (1.48-3.21) | <0.001^*^ |
| GBP5 | 1.21 (0.87-1.70) | 0.247 | 1.43 (1.02-2.00) | 0.035^*^ | 2.09 (1.35-3.23) | 0.001^*^ | 2.43 (1.65-3.58) | <0.001^*^ |

OS, overall survival; PFS, progression-free survival; HR, hazard ratio; CI, confidence interval.
