# Supplementary material for: M2 macrophage infiltration associated with CXCL8 predicts grade 4 prognosis and differentiates glioma grades
Source: Discov Oncol. 2025 Dec 23;16:2215. doi: 10.1007/s12672-025-03982-2 (PMC12728105; doi:10.1007/s12672-025-03982-2)
Supplement: Supplementary file 2 — Supplementary Material 2 [file 12672_2025_3982_MOESM2_ESM.docx]

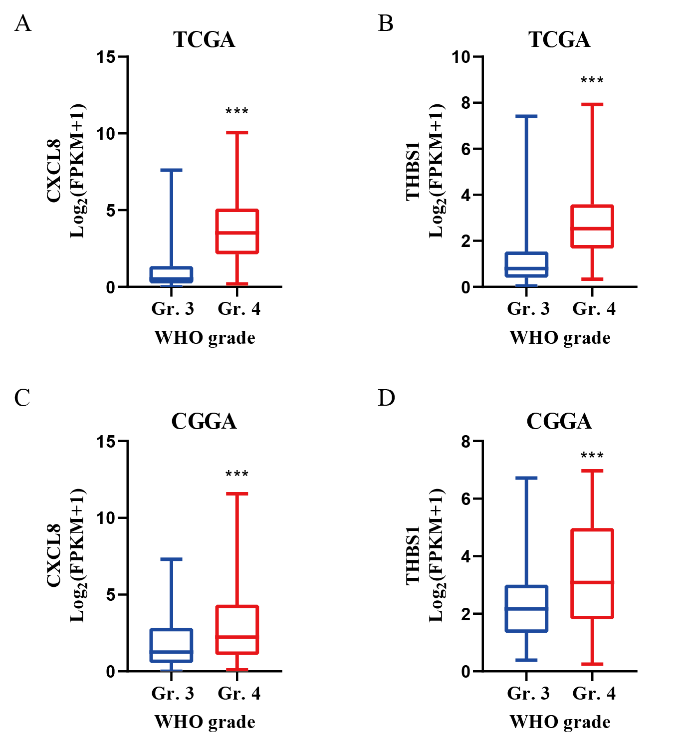

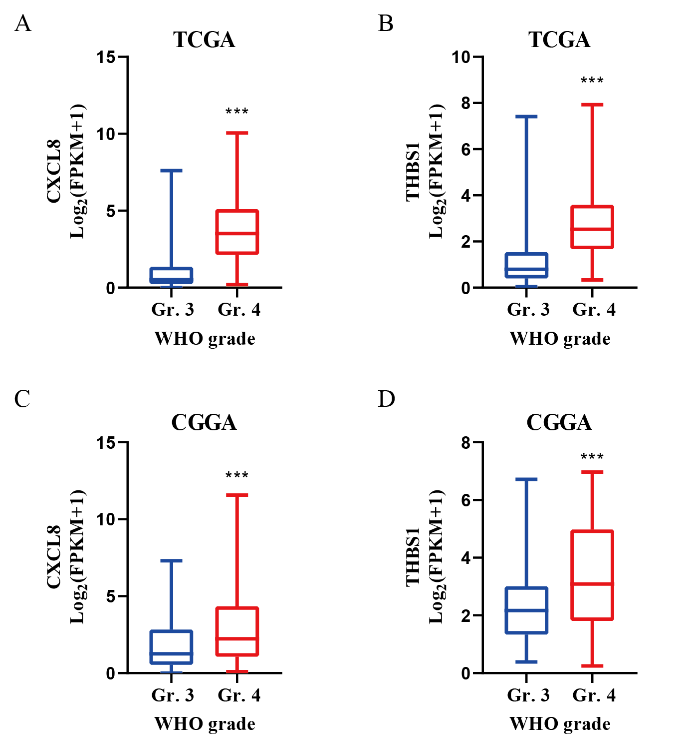

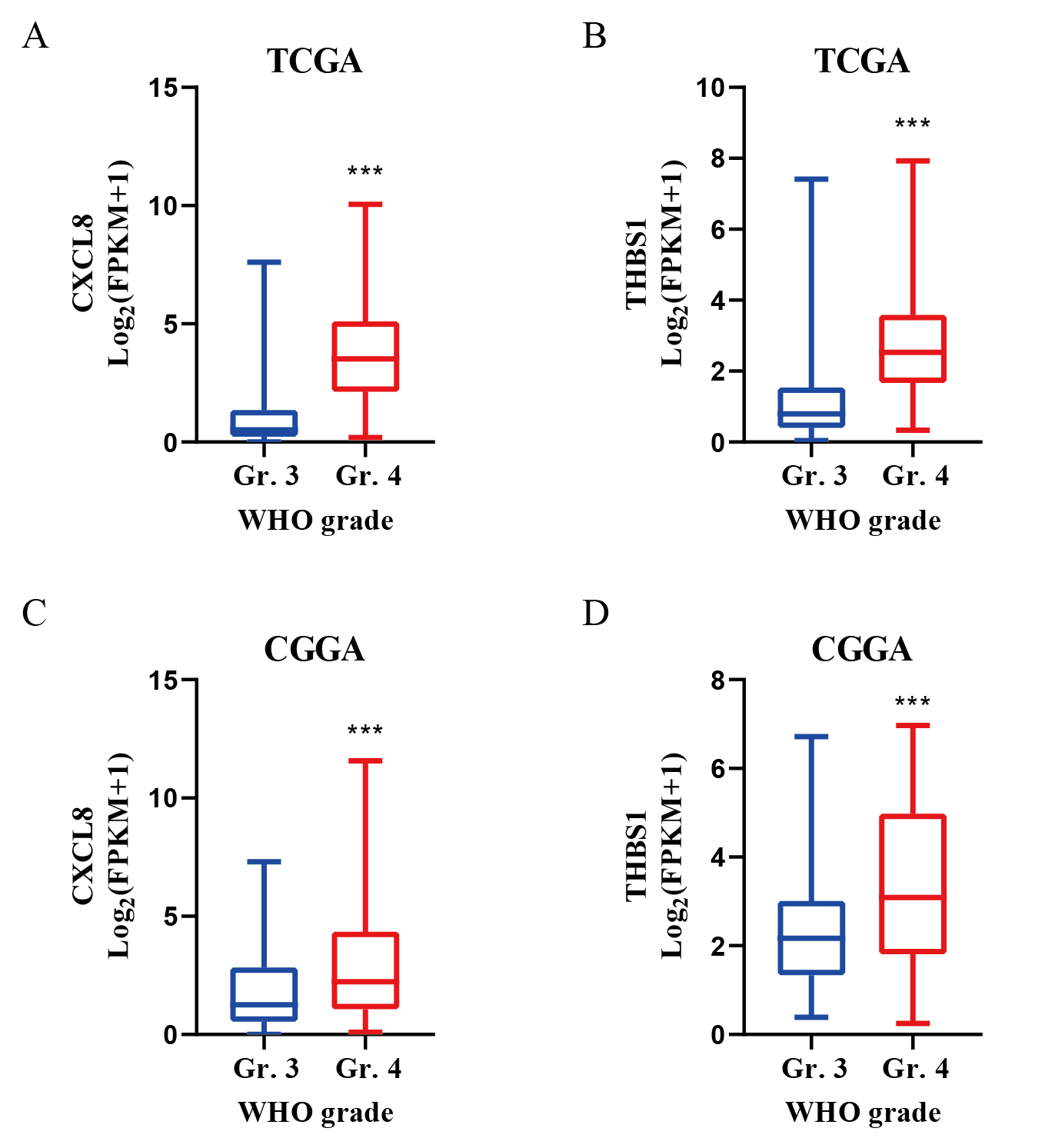

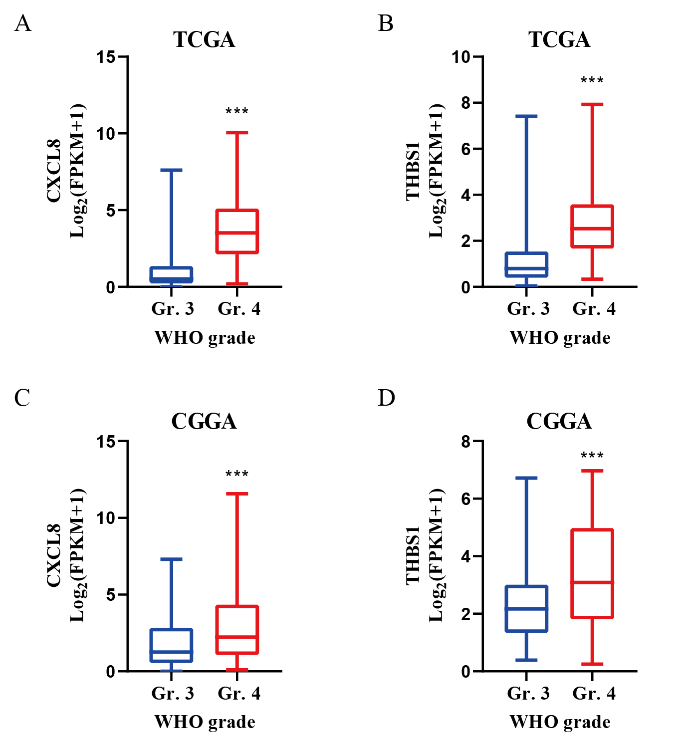


**Supplementary Figure S1.** RNA-seq data (FPKM) from TCGA and CGGA, and compared the gene expression levels of CXCL8 and THBS1 between Gr. 3 and 4; p-value <0.001 (∗∗∗).


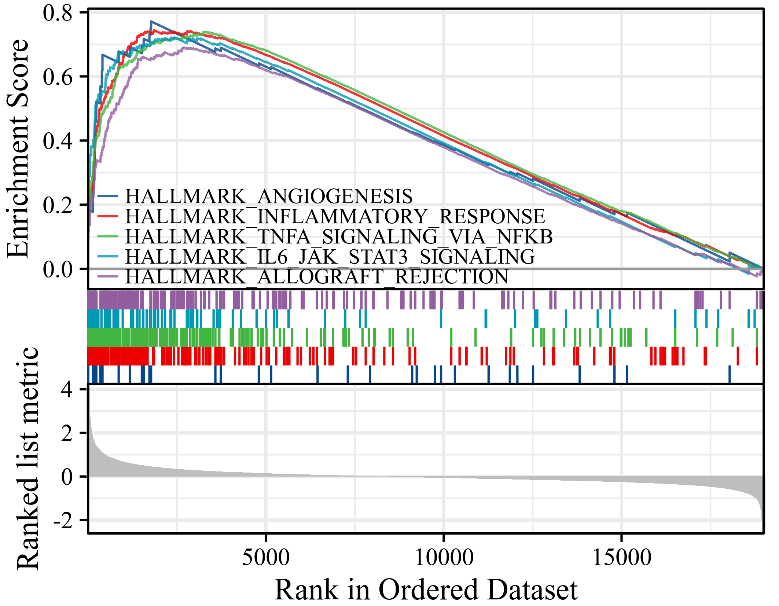
**Supplementary Figure S2.** Functional enrichment analysis of CXCL8 in Gr. IV glioma (GBM). (A) GSEA analysis between high (top 50%) - and low (bottom 50%)- CXCL8 expression using the TCGA GBM data. Based on enrichment scores, the angiogenesis pathway and immune-related pathways are enriched in groups of patients expressing high levels of CXCL8.

**Reference of supplement**

[1] Szklarczyk D, Franceschini A, Wyder S, Forslund K, Heller D, Huerta-Cepas J, et al. STRING v10: protein-protein interaction networks, integrated over the tree of life. Nucleic Acids Res. 2015;43:D447–D52.

[2] Shannon P, Markiel A, Ozier O, Baliga NS, Wang JT, Ramage D, et al. Cytoscape: a software environment for integrated models of biomolecular interaction networks. Genome Res. 2003;13:2498–504.

[3] Yu G, Wang LG, Han Y, He QY. clusterProfiler: an R package for comparing biological themes among gene clusters. Omics : a journal of integrative biology. 2012;16:284-7.

[4] Ogata H, Goto S, Sato K, Fujibuchi W, Bono H, Kanehisa M. KEGG: Kyoto encyclopedia of genes and genomes. Nucleic Acids Res. 1999;27:29–34.
